# Whole genome sequencing in the diagnosis of primary ciliary dyskinesia

**DOI:** 10.1186/s12920-021-01084-w

**Published:** 2021-09-23

**Authors:** Gabrielle Wheway, N. Simon Thomas, Mary Carroll, Janice Coles, Regan Doherty, Genomics England Research Consortium, Patricia Goggin, Ben Green, Amanda Harris, David Hunt, Claire L. Jackson, Jenny Lord, Vito Mennella, James Thompson, Woolf T. Walker, Jane S. Lucas

**Affiliations:** 1 School of Human Development and Health, Faculty of Medicine, University of Southampton, Southampton, UK; 2 Institute for Life Sciences, University of Southampton, Southampton, UK; 3 Wessex Regional Genetics Laboratory, Salisbury NSF Foundation Trust, Salisbury District Hospital, Salisbury, UK; 4 Wessex Clinical Genetics Service, University Hospitals Southampton NHS Foundation Trust, Southampton, UK; 5 School of Clinical and Experimental Sciences, Faculty of Medicine, University of Southampton, Southampton, UK; 6 Primary Ciliary Dyskinesia Centre, NIHR Biomedical Research Centre, University Hospital Southampton NHS Foundation Trust, Southampton, UK; 7 Biomedical Imaging Unit, University Hospital Southampton NHS Foundation Trust, Southampton, UK; 8 Queen Alexandra Hospital, Portsmouth Hospitals NHS Trust, Portsmouth, UK

**Keywords:** Primary ciliary dyskinesia, Non-CF bronchiectasis, Whole genome sequencing, Diagnosis, Gene discovery

## Abstract

**Background:**

It is estimated that 1–13% of cases of bronchiectasis in adults globally are attributable to primary ciliary dyskinesia (PCD) but many adult patients with bronchiectasis have not been investigated for PCD. PCD is a disorder caused by mutations in genes required for motile cilium structure or function, resulting in impaired mucociliary clearance. Symptoms appear in infancy but diagnosis is often late or missed, often due to the lack of a “gold standard” diagnostic tool and non-specific symptoms. Mutations in > 50 genes account for around 70% of cases, with additional genes, and non-coding, synonymous, missense changes or structural variants (SVs) in known genes presumed to account for the missing heritability.

**Methods:**

UK patients with no identified genetic confirmation for the cause of their PCD or bronchiectasis were eligible for whole genome sequencing (WGS) in the Genomics England Ltd 100,000 Genomes Project. 21 PCD probands and 52 non-cystic fibrosis (CF) bronchiectasis probands were recruited in Wessex Genome Medicine Centre (GMC). We carried out analysis of single nucleotide variants (SNVs) and SVs in all families recruited in Wessex GMC.

**Results:**

16/21 probands in the PCD cohort received confirmed (n = 9), probable (n = 4) or possible (n = 3) diagnosis from WGS, although 13/16 of these could have been picked up by current standard of care gene panel testing. In the other cases, SVs were identified which were missed by panel testing. We identified variants in novel PCD candidate genes (*IFT140* and *PLK4*) in 2 probands in the PCD cohort. 3/52 probands in the non-CF bronchiectasis cohort received a confirmed (n = 2) or possible (n = 1) diagnosis of PCD. We identified variants in novel PCD candidate genes (*CFAP53* and *CEP164*) in 2 further probands in the non-CF bronchiectasis cohort.

**Conclusions:**

Genetic testing is an important component of diagnosing PCD, especially in cases of atypical disease history. WGS is effective in cases where prior gene panel testing has found no variants or only heterozygous variants. In these cases it may detect SVs and is a powerful tool for novel gene discovery.

**Supplementary Information:**

The online version contains supplementary material available at 10.1186/s12920-021-01084-w.

## Background

Bronchiectasis is a disorder of persistent or recurrent bronchial infection, chronic cough, and purulent sputum production, associated with permanent airway dilatation. It is the end-point of various diseases and pathological mechanisms, and diagnosing bronchiectasis should therefore be the starting point to finding the underlying cause. Globally, severe lung infections such as tuberculosis, whooping cough, or measles can damage the airways at the time of infection, predisposing to a cycle of inflammation, structural airway damage and further infection culminating in irreversible bronchiectasis. In most Western populations, genetic conditions including cystic fibrosis (CF), primary ciliary dyskinesia (PCD) and primary immunodeficiency disorders are common causes of bronchiectasis. CF, caused by mutations in the *CFTR* gene is the most common genetic cause (≈1:2500 European Caucasians), and is usually picked up before bronchiectasis is established by new-born screening where it is available. PCD is less common (≈1:10,000 Caucasians, although higher in consanguineous populations eg 1:2200 in British Asians [1]), and despite having symptoms from infancy is often diagnosed in late childhood or adulthood [2]. The reasons for missed or late diagnoses are multi-factorial including no “gold standard” diagnostic tool, insufficient specialist diagnostic centres and clinicians’ lack of awareness about PCD [3]. PCD symptoms of daily wet cough, persistent rhinorrhoea, sinusitis and serous otitis media are non-specific. Patients with dextrocardia, which is rare in the general population but affects 50% of people with PCD, are diagnosed earlier than those with normal organ situs [2].

A combination of investigations is usually required to make a diagnosis of PCD [4, 5]. Whilst transmission electron microscopy (TEM) or genetics testing can confirm the diagnosis, each will miss 20–30% of patients, and other tests are therefore important [4, 6]. Whilst molecular genetic investigations play an essential role in the diagnosis of PCD, genetic results can be difficult to interpret without confirming the phenotype by TEM, immunofluorescent labelling of ciliary proteins and/ or highspeed video microscopy of ciliary beat pattern [7–9]. It is important to note that when all test results are ‘normal’, PCD cannot be ruled out conclusively albeit high-unlikely.

Mutations that cause PCD have been reported in > 50 genes [10]. These account for around 70% of cases, with additional genes, and non-coding, synonymous, missense changes or structural variants (SVs) in known genes presumed to account for the missing heritability. Many adult patients with non-CF bronchiectasis have not been investigated for PCD although it is estimated that 1–13% of cases of bronchiectasis in adults worldwide are attributable to PCD [11]. This wide range is due to different screening tools and diagnostic tests being used to diagnose PCD, and so it is difficult to assess the true rate of PCD in the adult bronchiectasis community. In the UK, patients suspected to have PCD can be sent for gene panel testing on a panel of known PCD disease genes alongside functional and structural tests of clia. This original panel consisted of 19 genes and later 29 genes (Additional file 1: Table S1). Whole genome sequencing (WGS) provides a second line opportunity for identification of non-coding changes and SVs in known PCD genes which may be missed by standard gene panel testing, and also provides opportunities for novel disease gene identification. UK patients with no genetic confirmation for the cause of their PCD or bronchiectasis were eligible for WGS in the UK 100,000 Genomes Project. The 100,000 Genomes Project, coordinated by Genomics England Ltd, aimed to deliver clinical benefits to patients by providing genetic diagnoses for people with cancer and rare diseases, with an additional and critically important spin off of providing a large amount of data for research. Furthermore this hybrid clinical and research project aimed to integrate WGS into the UK National Health Service (NHS), and to create the National Genomic Medicine Service [12, 13]. Recruitment to the project was coordinated across 13 Genome Medicine Centres (GMCs). Wessex GMC, covering Southampton University Hospital NHS Foundation Trust, a specialist PCD diagnostic centre, and Queen Alexandra Hospital, Portsmouth was the main recruiting GMC for non-CF bronchiectasis and the second largest recruiter of PCD probands in the 100,000 Genomes Project. We carried out clinical review of all variants returned from PCD and non-CF bronchiectasis patients by the 100,000 Genomes Project. This involved clinical variant classification following guidelines from the American College of Medical Genetics (ACMG) and the Association for Molecular Pathology (AMP) [14] and the current ACGS guidelines [15] to determine whether a variant was pathogenic, likely pathogenic, a variant of uncertain clinical significance, benign or likely benign. Genome sequence files, alignment files, variant call files and tiering files were also made available to approved researchers in a secure online research environment and we carried out comprehensive independent research analysis of these files for patients recruited in Wessex GMC, with an aim to identify pathogenic variants missed by the automated Genomics England Ltd tiering process, and uplift genetic diagnosis in these cohorts.

## Methods

### Patient selection and recruitment to the 100,000 Genomes Project

Recruitment to the 100,000 Genomes Project was initially restricted to patients who had undergone prior genetic diagnostic testing and had not received a genetic diagnosis, although later in the project this was relaxed to include patients who had not undergone previous genetic testing. The PCD cohort (n = 42, of which probands n = 21) comprised patients from the PCD Adult and Paediatric Centre at University Hospital Southampton (UHS) who had no genetic diagnosis of PCD, and their first-degree relatives. 9/21 probands had no prior genetic testing. 1/21 probands had previously had targeted sequencing of *DNAH11*, 7/21 had been tested against a gene panel which included 19 known PCD genes (Additional file 1: Table S1), and 4/21 had previously been tested against a gene panel which included 29 known PCD genes (Additional file 1: Table S1). Of the 12 patients with prior testing, 9 had variants identified but these were either heterozygous (2/12) or did not confirm a genetic diagnosis (because of 1 or more variants were variants of uncertain clinical significance (VUS)). These patients were recruited for WGS as part of 100,000 Genomes Project. The non-CF bronchiectasis cohort (n = 69, of which probands n = 52) included patients under adult pulmonology care at UHS and Portsmouth Hospitals NHS Trust. Informed consent was obtained for participation in the 100,000 Genomes Project, and where appropriate for PCD diagnostic testing (National Research Ethics Service South Central (A) Committee 07/Q1702/109 and University of Southampton Faculty of Medicine Ethics Committee ERGO#53,155). Clinical characteristics were recorded using Human Phenotype Ontology (HPO) terms and blood taken for DNA extraction.

### Diagnostic testing in Southampton PCD diagnostic service

PCD diagnostic testing was undertaken for all participants in the PCD cohort prior to recruitment to 100,000 Genomes Project. Participants in the non-CF bronchiectasis cohort were invited for PCD functional testing and biomedical imaging if the WGS found variants in PCD-related genes. A detailed clinical history was recorded using a proforma. PCD diagnostic testing included nasal nitric oxide (nNO), high-speed video microscopy (HSVM) analysis [8], transmission electron microscopy (TEM) and immunofluorescence (IF) microscopy imaging, as previously described [8, 16–19]. In brief, nNO was measured by chemiluminescence analyser (Ecomedics CLD 88 Exhalyzer) during exhalation against resistance with sampling at 0.33 l/min and a cut-off of 77nL/min. High-speed video analysis was conducted in a 37 °C heated environmental chamber; images were digitally recorded using a high-speed camera at a rate of 500 frames per second (fps) and reviewed at reduced frame rates (30–60 fps) for analysis of ciliary beat pattern (CBP) and ciliary beat frequency (CBF). To differentiate primary from secondary dyskinesia samples were reanalysed following in vitro differentiation of basal epithelial cells and ciliation in an air–liquid interface culture [20]. 100–300 cilia were imaged by Hitachi H7000 electron microscope in transverse section for qualitative and quantitative assessment of axonemal structure (minimum magnification 60,000×) [19]. Immunofluorescent confocal imaging of targeted PCD related proteins used a Leica SP8 confocal microscope to identify mislocalised or absent proteins [21]. Antibodies used included RPGR Sigma HPA001593 (1:200), Alpha tubulin clone DM1A Sigma T9026 (1:500), Alexafluor 488 Invitrogen A21121 (1:2500) and Alexafluor 594 Invitrogen A11012 (1:2500). Confocal immunofluorescence images of nasal brushings and ALI cultures were taken on a Leica TCS-SP8 laser scanning confocal system with true spectral detection attached to a Leica DMI8 inverted microscope frame and using Leica LAS-X v3.5.7 acquisition software. All images were taken using a Leica HC PL APO CS2 63x / 1.3 N.A. glycerol immersion lens as single Z slices at a visually-optimised focal plane. Imaging parameters were 1024 × 1024 pixel sampling (XY), 600 Hz scan speed, 4 line averaging and -1% offset for background correction and 3 × optical zoom (by restricting angular deflection of the scanning mirrors), providing nyquist sampling at a 80 nm pixel size (XY). Sequential imaging (DAPI + AF594/AF488) was used to avoid spectral bleed though between channels. The photomultiplier gain for the RPGR antibody was set to a constant level for all the images. No post processing of images was performed.

### Whole genome sequencing and data analysis

**Fig. 1:**
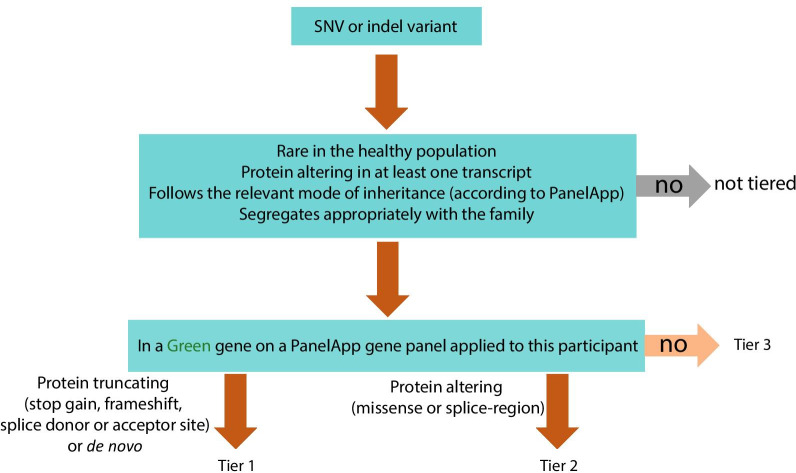
Genomics England Ltd (Genomics England Ltd) variant filtering and tiering strategy for single nucleotide variants (SNVs) or small insertions or deletions. Tier 1 and tier 2 variants were returned to recruiting Genome Medicine Centres (GMC) for variant review and classification following American College of Medical Genetics guidelines. Tier 3 variants were available for review in the research environment. Primary ciliary disorders PanelApp gene panel can be found in Table 1, non-CF bronchiectasis PanelApp gene panel can be found in Table 2

A detailed description of the 100,000 Genomes Project protocol can be found at https://www.genomicsengland.co.uk/wp-content/uploads/2017/03/GenomicEnglandProtocol_151117-v4-Wales.pdf. In brief, DNA was extracted and stored in a central bioresource, PCR-free libraries prepared and sequenced using Illumina short-read technology, sequence data aligned to the human reference genome, followed by variant calling and annotation. Genomics England Ltd performed variant filtering through an automated analysis pipeline to identify potentially diagnostic small nucleotide variants (SNVs) and structural variants (SVs), which were “tiered” according to likelihood of being the pathogenic cause of disease if in clinically relevant genes (Tables 1, 2) [22] and consistent with the observed pattern of inheritance [12]. Tier 1 and 2 SNVs (Fig. 1) and all SVs > 10 kb (ie variants most likely to be clinically relevant) were returned to GMCs for clinical review and classification of variants following ACMG/AMP guidelines [14] and the current ACGS guidelines [15] to determine whether they were pathogenic, likely pathogenic, a variant of uncertain clinical significance, likely benign or benign. These were then reported back to recruiting clinicians and patients.

**Table 1:** Green genes on the primary ciliary disorders PanelApp virtual gene panel, considered diagnostic for PCD in the clinical analysis pipeline of the 100,000 Genomes Project

Gene Name	Model_Of_Inheritance	OMIM number
*ARMC4*	Autosomal recessive	615451
*C21orf59*	Autosomal recessive	615500
*CCDC103*	Autosomal recessive	614679
*CCDC114*	Autosomal recessive	615067
*CCDC151*	Autosomal recessive	616037
*CCDC39*	Autosomal recessive	613807
*CCDC40*	Autosomal recessive	613808
*CCDC65*	Autosomal recessive	615504
*CCNO*	Autosomal recessive	615872
*DNAAF1*	Autosomal recessive	613193
*DNAAF2*	Autosomal recessive	612518
*DNAAF3*	Autosomal recessive	606763
*DNAAF4*	Autosomal recessive	127700
*DNAAF5*	Autosomal recessive	614874
*DNAH11*	Autosomal recessive	611884
*DNAH5*	Autosomal recessive	608644
*DNAI1*	Autosomal recessive	244400
*DNAI2*	Autosomal recessive	612444
*DNAL1*	Autosomal recessive	614017
*DRC1*	Autosomal recessive	615294
*GAS8*	Autosomal recessive	616726
*HYDIN*	Autosomal recessive	608647
*LRRC6*	Autosomal recessive	614935
*MCIDAS*	Autosomal recessive	618695
*PIH1D3*	X-linked recessive	300991
*RPGR*	X-linked recessive	300455
*RSPH1*	Autosomal recessive	615481
*RSPH3*	Autosomal recessive	616481
*RSPH4A*	Autosomal recessive	612649
*RSPH9*	Autosomal recessive	612650
*SPAG1*	Autosomal recessive	615505
*ZMYND10*	Autosomal recessive	615444

Gene name, pattern of inheritance and OMIM ID are provided for each gene. This panel was originally 31 genes at the start of the project; **PIH1D3** was added towards the end of the project. All PCD patients had this panel applied. Some but not all non-CF bronchiectasis patients had this panel applied

**Table 2:** Green genes on the non-CF bronchiectasis PanelApp virtual gene panel, considered diagnostic for non-CF bronchiectasis and CF in the clinical analysis pipeline of the 100,000 Genomes Project

Gene Name	Model_Of_Inheritance	OMIM number
*CFTR*	Autosomal recessive	219700
*PIK3CD*	Autosomal dominant (not imprinted)	615513
*SCNN1A*	Autosomal dominant (imprinted status unknown)	613021
*SCNN1B*	Autosomal dominant, autosomal recessive	211400
*SCNN1G*	Autosomal dominant (not imprinted)	613071

Gene name, pattern of inheritance and OMIM ID are provided for each gene

In parallel, all anonymised sequence data, small nucleotide variant and structural variant files, HPO data and tiering data for each participant was made available for interrogation by approved researchers through the secure online Research Environment within the Genomics England Data Embassy. PCD and non-CF bronchiectasis research analysis was conducted on this data in the research environment. Further details can be found in Additional file 2.

### Clinical review and classification of variants

We reviewed all tier 1 and tier 2 variants (Fig. 1) and SVs > 10 kb using ACMG/AMP guidelines for clinical variant interpretation [14] and the current ACGS guidelines [15]. Where appropriate, variants were classified at a genomics MDT which included reviewing PCD diagnostic results where available. Although PCD is genetically heterogeneous, review of the PCD clinical diagnostic testing data (nNO, HSVM, TEM and IF) enabled the PP4 line of evidence to be applied to some specific cases (a supporting level of evidence that a variant is pathogenic can be applied when the patient’s phenotype, in this case cellular phenotype, is highly specific for the gene of interest). Where a SV was identified, the corresponding sequence data were manually interrogated. Sequence and coverage data were also manually inspected to verify the presence of variants identified SNVs were confirmed by Sanger sequencing or other parallel testing. SVs were confirmed by PCR amplification of the deletion breakpoint and Sanger sequencing of the PCR product.

Where tiered variants did not provide a diagnosis, we proceeded to use custom Python scripts to extract heterozygous SNVs in known PCD genes, and heterozygous and homozygous SNVs in candidate ciliopathy genes [23, 24], and SVs < 10 kb in all genes of interest using WGS data for all PCD and non-CF bronchiectasis probands recruited in Wessex GMC.

This analysis was carried out under approved Genomics England research project RR185, with ethical approval from University of Southampton Faculty of Medicine Ethics Committee (ERGO#54,400).

## Results

### PCD cohort participants

The 100,000 Genomes Project recruited 118 PCD patients nationally (274 individuals when including probands and family members). 21 PCD probands were recruited in Wessex GMC (41 participants including family members) including 9 singletons, 4 duos and 8 trios. We first describe the results returned in the clinical tiering analysis, and then the results of our independent research analysis.

### Results of SNV analysis in PCD cohort

Tier 1 or tier 2 variants were found in 12/21 patients. ACMG variant classification confirmed a diagnosis of PCD in 6 cases (biallelic pathogenic/likely pathogenic variants) (Table 3; cases 1–6). The causative genes were *DRC1, DNAI1*, *DNAH11* (× 2) and *DNAH5 (*× *2)*. Of the 6 confirmed cases, 4 had not undergone prior testing but the variants were all identifiable by standard panel testing. A diagnosis was now possible in the 2 cases that had undergone prior testing because either additional cases were available in the literature or parental testing demonstrated that the variants were *in trans* (allowing the PM3 line of evidence to be applied); WGS did not identify any additional variants.

**Table 3:**
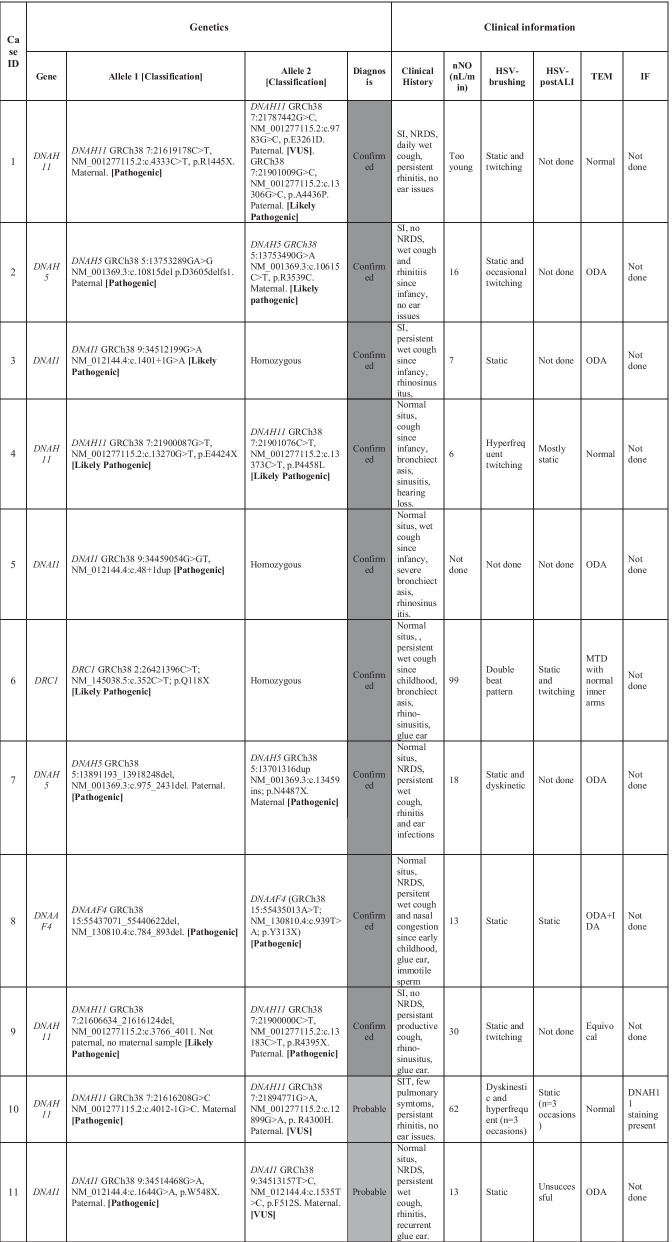

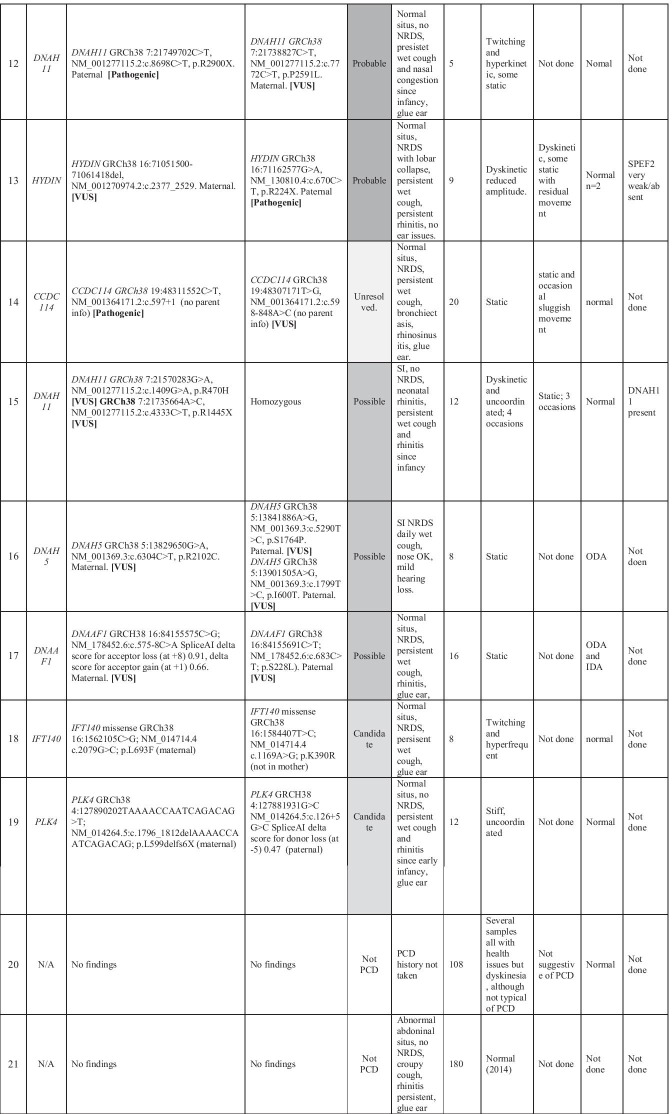
Genetic testing results from panel testing, 100,000 Genomes Project whole genome sequencing, clinical history and PCD diagnostic test results for each proband 1–21 in the Wessex PCD cohort

ACMG variant classification for each variant is given in bold

The table provides case ID, gene and variant details (with GRCh38 genomic coordinates) and classification (following ACMG guidelines) and final diagnosis. Clinical information provided includes clinical history, nasal nitric oxide levels (nNO (nL/min)), high-speed video (HSV) microscopy analysis from fresh nasal brushings and afterculture of these cells at air–liquid interface (ALI) transmission electron microscopy (TEM) and immunofluorescence (IF) microscopy findings. SI = *Situs inversus*, NRDS = neonatal respiratory distress syndrome, ODA = outer dynein arm defect, IDA = inner dynein arm defect, MTD = Microtubular disorganisation

A further 3 cases received a probable diagnosis (biallelic pathogenic or likely pathogenic variant and VUS in a known PCD gene) (Table 3; cases 10–12). The variants were in *DNAI1* and *DNAH11* (× 2). All had undergone prior targeted *DNAH11* or gene panel testing and received the same findings. Re-analysis of the VUS did not result in reclassification, and so the diagnosis remains as probable but not confirmed. WGS did not provide useful additional information in these cases but confirmed findings from gene panel testing.

A further 3 cases received a possible diagnosis (biallelic VUS(s) in a known PCD gene) (Table 3; 15–17). Two of these had undergone prior gene panel testing and received the same result. The genes were *DNAAF1*, *DNAH5* and *DNAH11.* Again, re-analysis of the VUS did not result in reclassification and so the diagnosis remains as possible but not confirmed. One case had not undergone prior testing, and so received a new result, but this could have been obtained through gene panel testing rather than WGS.

### Results of SV analysis in PCD cohort

SV > 10 kb analysis identified a heterozygous pathogenic SV of around 27 kb in *DNAH5*, deleting exons 8–16 (inclusive) in one family. Manual inspection of *DNAH5* sequence data identified a stop gain *in trans* with the SV providing an additional confirmed diagnosis (Table 3; case 7). This heterozygous stop gain variant was not tiered (due to being heterozygous) and so this diagnosis could have been missed by the Genomics England Ltd pipeline. This participant had undergone prior gene panel testing which identified the heterozygous *DNAH5* stop gain variant but not the SV. In this case WGS was useful in providing confirmed genetic diagnosis in this individual which was not possible with gene panel testing.

In summary, 13/21 participants received confirmed, probable or possible diagnostic results from the initial clinical analysis of tiered data and SVsGenomics England Ltd. The causative genes were *DNAAF1, DRC1, DNAI1* (× 3), *DNAH11* (× 5) and *DNAH5* (× 3). These were new findings in 6/13 cases and in 7/13 cases confirmed results from prior panel testing.

8/21 participants did not receive a result from the initial analysis of tiered variants.

**Fig. 2:**
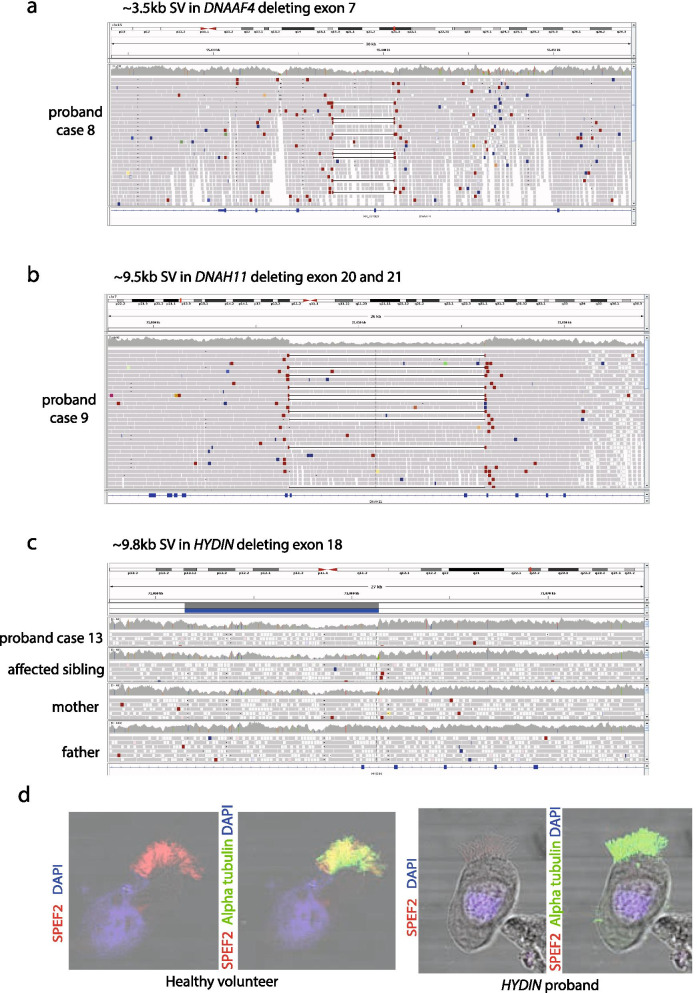
Structural variants in known PCD genes identified in unsolved patients in the Wessex PCD cohort. IGV screenshot showing heterozygous ~ 3.5 kb SV in *DNAAF4* deleting exon 7 in case 8 in Table 3. This was found *in trans* with a stop gain variant in *DNAAF4*, providing a confirmed diagnosis for this patient. **a** IGV screenshot showing heterozygous ~ 9.5 kb SV in *DNAH11* deleting exon 20 and 21 in case 9 in Table 3. This results in an in-frame deletion of amino acids 1256–1337 containing a coiled-coil domain which may take part in protein–protein interactions. Multiple alignment of DNAH11 protein sequence across multiple species shows that this region of the protein is well conserved and likely to be functionally important, so that the deletion would be deleterious to protein function. The ACMG classification was likely pathogenic. This was found *in trans* with a pathogenic stop gain variant in *DNAH11*, providing a confirmed diagnosis for this patient. **b** IGV screenshot showing heterozygous ~ 9.8 kb SV in *HYDIN* deleting exon 18 in case 13, affected sibling and mother but not father. This results in an in-frame deletion of amino acids 793–843 of the 5121 amino acid protein. Multiple alignment of HYDIN protein sequence across multiple species shows that this region of the protein is conserved and likely to be functionally important, but the ACMG classification was variant of uncertain significance because we couldn’t show that the deleted interval was critical to protein function. This was found *in trans* with a stop gain variant in *HYDIN* (also found in affected sibling and father), providing a probable diagnosis for this patient and their affected sibling. **c** Immunofluorescence confocal image of SPEF2 (red), ciliary axoneme marker alpha tubulin (green) and DAPI, overlaid with phase contrast image of single multiciliated cells from the nasal epithelium of health volunteer (left) and PCD case 13, who has a 9.8 k deletion in HYDIN in trans with a stop gain variant in HYDIN. HYDIN is required for trafficking of SPEF2 into the cilia, and so absence of SPEF2 in the cilia of this patient is supportive of the genetic findings in this patient

From the 8 negative cases, we proceeded to extract and study heterozygous SNVs in known PCD genes, and found 4 heterozygous potentially pathogenic stop gain or splice site variants (Table 3; cases 8–9 + 13–14) in *DNAAF4, DNAH11, HYDIN* and *CCDC114*. One of these had undergone prior gene panel testing that had identified the single heterozygous SNV in *DNAAF4*. We then visually analysed the alignment files of these participants in IGV to identify any SVs. This identified that 3 of the cases had biallelic SNVs and SVs in PCD genes which were not picked up by the tiering pipeline, as they were < 10 kb):

- stop gain +  ~ 3.5 kb SV in *DNAAF4* deleting exon 7 (Table 3; case 8, Fig. 2a)
- stop gain +  ~ 9.5 kb SV in *DNAH11* deleting exon 20 and 21 (Table 3; case 9, Fig. 2b)
- stop gain +  ~ 9.8 kb SV in *HYDIN* deleting exon 18 (Table 3; case 13, Fig. 2c)

The *DNAAF4, DNAH11* and *HYDIN* SNVs and SVs were found to segregate appropriately in the family and were all classified as pathogenic following ACMG guidelines, except the *HYDIN* SV which was classified as VUS. This provides 2 further confirmed diagnoses and 1 probable diagnosis. We were satisfied that *HYDIN* was the causative gene by observing absence of SPEF2 in the motile cilia of the proband, shown by IF confocal imaging (Fig. 2d). HYDIN is required for trafficking of SPEF2 into the cilia, and so absence of SPEF2 in the cilia of this patient is supportive of the genetic findings in this patient [25]. However, due to issues around poor mapping quality caused by *HYDIN* pseudogene, *HYDIN2*, we plan to carry out additional RNA investigations to further corroborate these genetic findings. We are confident that the stop gain is in *HYDIN* as sequence reads show it is in *cis* with an intronic SNV that is divergent between *HYDIN* and *HYDIN2* but wish to confirm that the SV is specific to *HYDIN*. The two *DNAH11* findings were novel, this patient did not undergo prior testing. The two *HYDIN* findings were novel, despite this patient having undergone 29 gene panel testing previously. In these cases WGS was extremely useful in providing confirmed genetic diagnosis in these individuals. It is unlikely that these SVs could have been detected by the small custom PCD gene panel testing.

No SVs were found in *CCDC114,* but we did find a novel deep intronic variant in *CCDC114* which is absent from gnomAD and may affect splicing, although this is not predicted by SpliceAI [26]. Segregation analysis was not possible in this case, as the proband was recruited as a singleton. Furthermore, this individual had normal ciliary ultrastructure by TEM, but outer dynein arm defects would be expected in a *CCDC114* patient. The official ACMG classification of this intronic variant is variant of uncertain significance (VUS), and with the second pathogenic SNV in *CCDC114* this provides a probable diagnosis following ACMG guidelines. However, ERS PCD Diagnostic guidelines say “Genetic diagnosis has to be consistent with the clinical and TEM/IF/HSVA phenotype, or diagnosis reconsidered if the picture is inconsistent” and so, on balance, we class this as unresolved, with additional work (RNA studies) required to further investigate this case.

This left 4/21 additional unsolved PCD cases, 2 of whom had undergone prior gene panel testing. In these cases we studied tier 3 variants, applying a functional candidate gene approach by using an updated version of the Syscilia Gold Standard list of known cilia genes [23] and CiliaCarta list of candidate cilia genes [24]. This identified biallelic variants in two cilia or centrosomal genes which have been linked to other OMIM diseases but not PCD (Table 3; cases 18 + 19): *IFT140*, which has been reported in isolated retinitis pigmentosa (RP) [27, 28] and syndromic RP [29] and several short rib polydactyly syndromes [30–32] and *PLK4* which has been associated with microcephaly and chorioretinopathy [33]. The *IFT140* variants are both missense variants; a rare p.(Leu693Phe) which is predicted by PolyPhen to be tolerated and by SIFT to be benign, and a rare p.(Lys390Arg) which is predicted by PolyPhen to be tolerated and by SIFT to be benign. The *PLK4* variants are a frameshift loss-of-function and a -5 splice variant which is predicted to cause loss of the splice donor site, with a SpliceAI donor loss delta score of 0.47 (delta scores > 0.5 are reliable predictors of a biological effect on splicing). In order to validate *PLK4* as the causative gene, RNA analysis will need to be carried out. In these patients, WGS will have proved invaluable if our further research work can confirm diagnoses in these cases. In the remaining two cases no candidate variants were identified and re-review of the clinical phenotype information indicated that historical diagnostics were not suggestive of PCD, and these patients were inappropriately recruited within the PCD disease category. In these cases WGS analysis was useful in confirming that these patients had no genetic cause of PCD, although this could have been concluded through re-review of their clinical notes.

## Summary of WGS results from patients recruited in PCD cohort

**Fig. 3:**
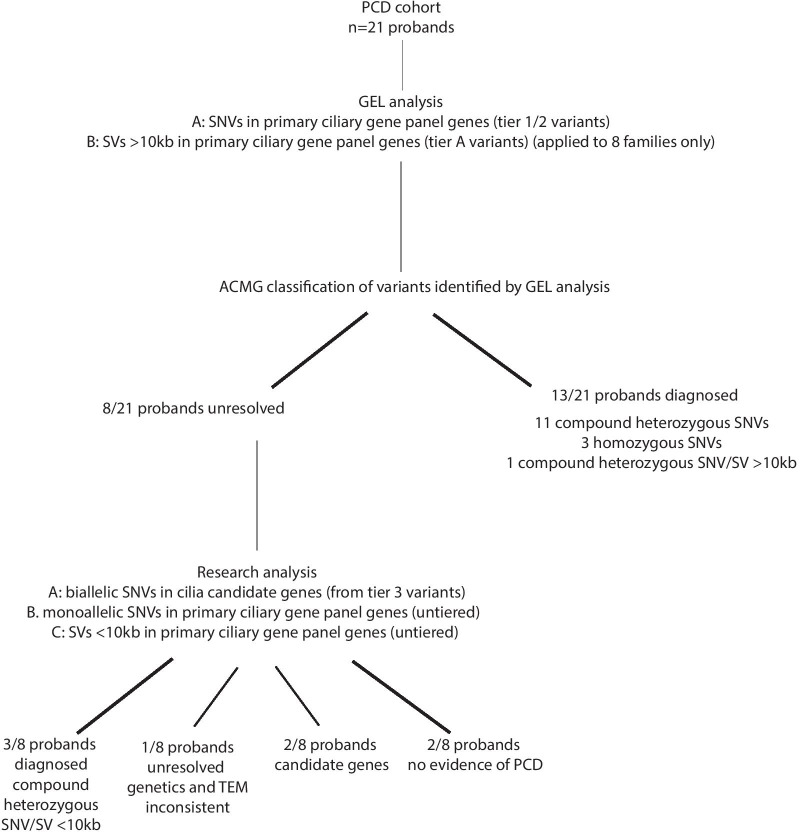
Flowchart showing clinical and research analyses carried out and all results for Wessex PCD cohort

**Fig. 4:**
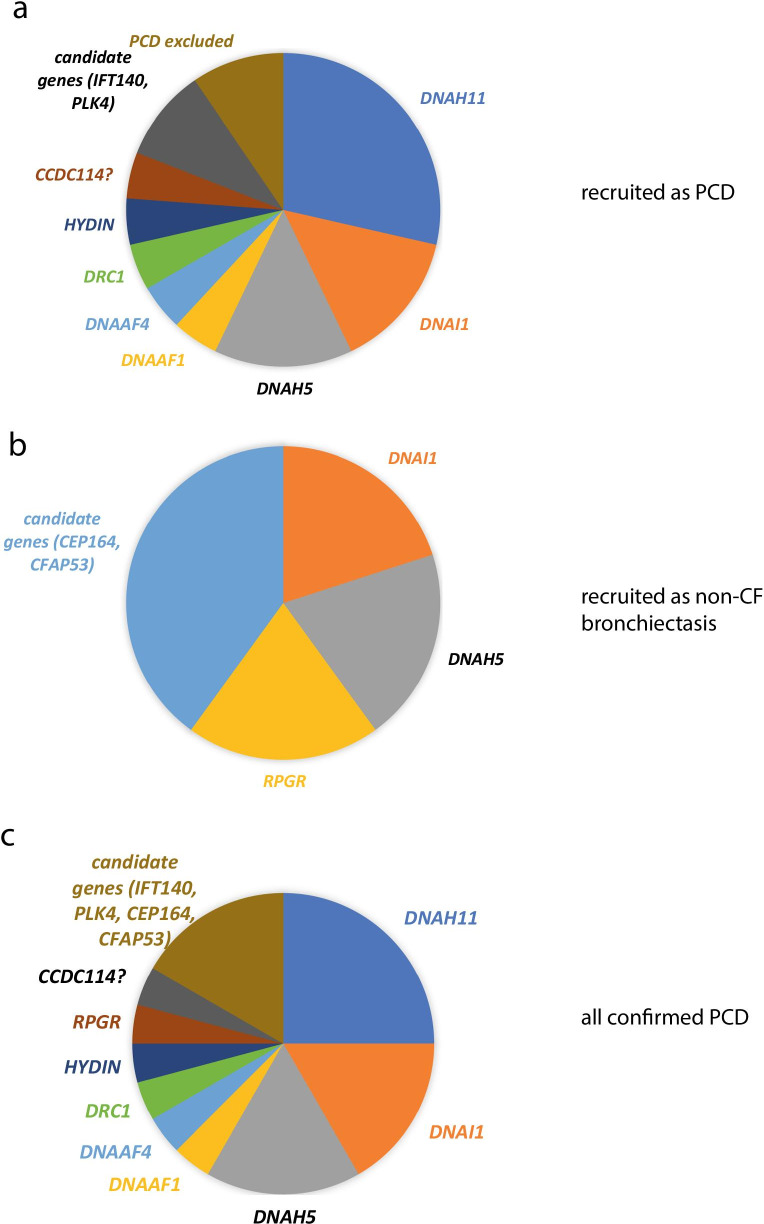
Causative genes in PCD cases recruited as PCD or recruited as non-CF bronchiectasis. **a** Pie chart chowing proportion of patients recruited into PCD disease category with causative variants in different known and candidate PCD genes. **b** Pie chart chowing proportion of patients recruited into non-CF disease category with causative variants in different known and candidate PCD genes. **c** Pie chart chowing proportion of patients recruited into PCD and non-CF disease categories with causative variants in different known and candidate PCD genes

In total 16 participants received confirmed, probable or possible genetic diagnosis from all analyses (Fig. 3). In all cases the diagnostic phenotype, including cellular and ciliary phenotype, was consistent with the genotype. One additional case had genetic findings sufficient for a probable diagnosis (ie Pathogenic variant + VUS) following ACMG classification, but this was at odds with TEM findings; the variants were in *CCDC114*, where ODA defects would be expected on TEM but were not seen. Following ERS PCD diagnostic guidelines this case remains unresolved. 13 of the diagnoses were provided by Genomics England Ltd tiering analysis and 3 were obtained through further research analysis of the WGS data. 3/16 patients had homozygous SNVs, 13/16 patients were compound heterozygous and in 4 of these patients one of the variants was a SV. Only 1 of these SVs was reported by Genomics England Ltd, as it was > 10 kb. The other 3 were found manually and were all < 10 kb. Furthermore, research analysis identified variants in candidate PCD genes (*IFT140* and *PLK4*) in 2 cases. Thorough genetic analysis and diagnostic imaging found no evidence of PCD in 2 cases. In the 16 cases which received a diagnosis, the causative genes were: *DNAAF1*, *DNAAF4*, *DRC1, DNAI1* (× 3), *DNAH11* (× 6), *DNAH5* (× 3) and *HYDIN* (Fig. 4). As variants in *DRC1* have not been reported in many patients, we report the clinical features of the *DRC1* patient here.

### *DRC1* case study

A 34-year-old man from a non-consanguineous background presented with a persistent wet cough since childhood, sino-rhinitis, glue ear and bronchiectasis. He had a PICADAR score [34] of 4/14 which is below the level recommended for referral for diagnostic testing. Nasal nitric oxide (nNO) levels were within the normal range (CLD 88 Exhalyzer; exhalation against resistance; 109 nl/min). High speed video microscopy (HSVM) of nasal epithelial cells showed uncoordinated cilia with an intermittent double beat ‘tick’ and limited movement. The beat pattern was persistently abnormal on four different occasions, and also following culture at air liquid interface; according to international guidelines this is not diagnostic but makes a positive diagnosis highly likely [4]. TEM showed 20% of cilia displayed microtubular disorganisation (MTD) which in itself is insufficient to diagnose PCD, but might suggest mutations in *CCDC65, DRC1* or *GAS8* encoding nexin-dynein regulatory complex proteins [19]. Genetic testing as part of a research programme in 2013 (not standard of care testing, therefore not recorded in Table 3) had failed to identify a causative gene, and six years later this project finally provided a definitive diagnosis of PCD caused by homozygous stop gain mutation in *DRC1 (CCDC164);* a gene described to cause PCD in 2013 [35].

Therefore, although functional and imaging tests were abnormal, they did not confirm a definite diagnosis of PCD. Using high speed video and TEM the abnormalities were subtle, likely to be missed by non-specialists. Indeed the literature sometimes describes high speed video and TEM as normal in patients with mutations in *DRC1*. In this patient nNO levels were normal, although they have previously been reported as abnormally low in patients with mutations in *DRC1*. We were not using immunofluorescent analyses at the time the patient was diagnosed, but would anticipate absence of GAS8 localisation in the cilia, as DRC1 and GAS8 are both components of the nexin-dynein regulatory complex (N-DRC) in the cilium and mutations in DRC1 cause N-DRC defects which can be observed as absence of GAS8 localisation from the cilium [36]. This case highlights the important role of genetic testing in variants where functional and imaging tests might be normal or equivocal, and also the need to revisit inconclusive cases as new PCD-causing genes are described.

### Non-CF bronchiectasis cohort participants

52 non-CF bronchiectasis probands were recruited in Wessex GMC (69 participants including family members), including 44 singletons. Between 2 and 9 different PanelApp panels were applied to these cases, with an average of 2.5 panels. None had prior genetic testing for PCD. We first describe the results of the analysis carried out by Genomics England Ltd and reviewed in the GMC, and then describe the results of our independent research analysis.

### Results of SNV analysis in non-CF bronchiectasis cohort

**Fig. 5:**
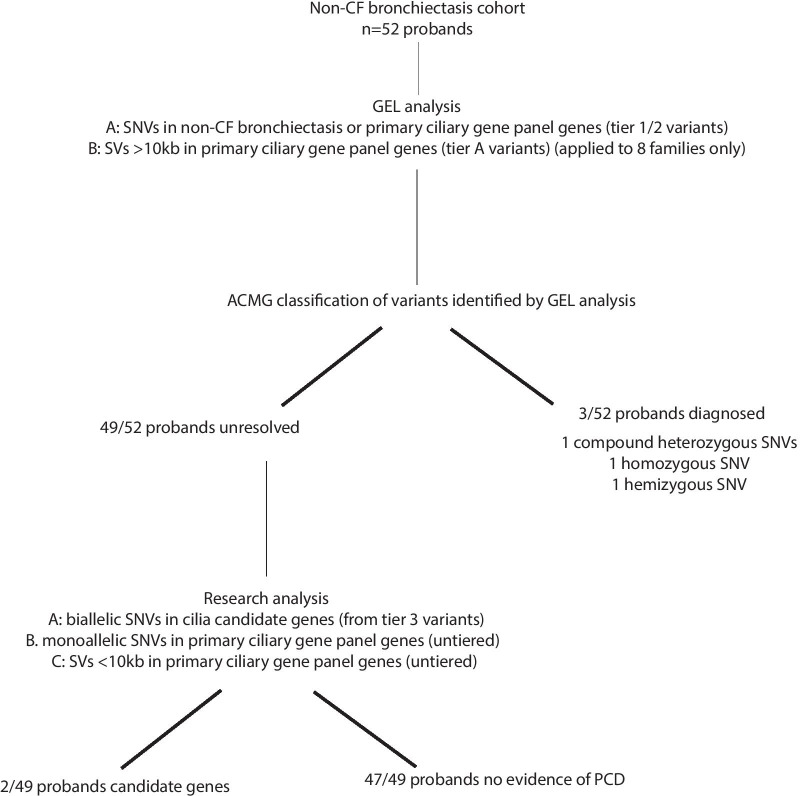
Flowchart showing clinical and research analyses carried out and all results for Wessex non-CF bronchiectasis cohort

**Fig. 6:**
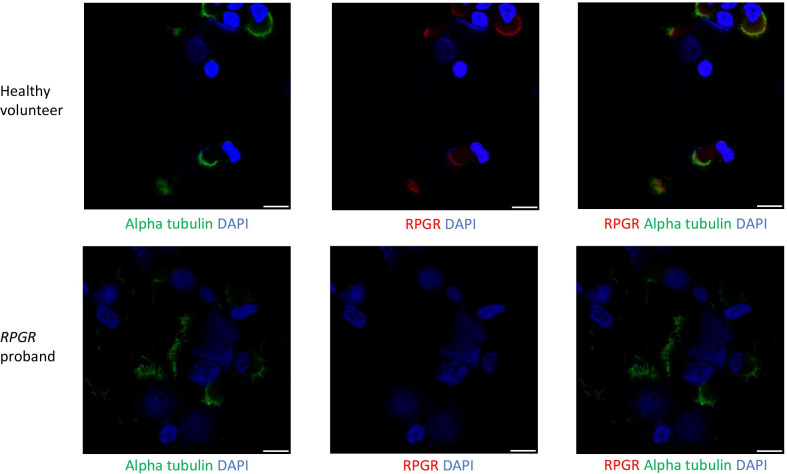
Immunofluorescence confocal images of multiciliated nasal epithelial cells from non-CF bronchiectasis patient with mutation in RPGR. Immunflourescence confocal images of RPGR (red), ciliary axoneme marker alpha tubulin (green) and DAPI in multiciliated cells from the nasal epithelium of health volunteer (left) and non-CF bronchiectasis case C, who has a predicted damaging hemizygous p.H201R missense mutation in RPGR. Absence of RPGR in the cilia of this patient is supportive of the genetic findings in this patient. Scale bar = 10 µm

In the initial analyses the PCD virtual gene panel was applied to only 37 families. 3 individuals had tier 1 or 2 variants in a PCD gene. After variant classification following ACMG guidelines, diagnosis of PCD was confirmed in 2/3 of these individuals (causative genes *DNA1* and *DNAH5*) and the diagnosis of PCD was possible in 1/3 individuals (causative gene *RPGR*) (Table 4, Fig. 5). Although *RPGR* is formally a VUS, IF result suggests it is the cause of the patient’s PCD (Fig. 6).

**Table 4:** Genetic testing results from 100,000 Genomes Project whole genome sequencing, clinical history and PCD diagnostic test results for each proband A-E in the Wessex non-CF bronchiectasis cohort found to have results consistent with a diagnosis of PCD

Anonymised family ID	Genetics				Clinical information					
	Gene	Allele 1 [Classification]	Allele 2 [Classification]	PCD Diagnosis	Clinical History	nNO (nL/min)	HSV-brushing	HSV-postALI	TEM	IF
A	*DNAH5*	*DNAH5* GRCh38 5:13780960C > A,NM_001369.3:c.8821-1C > A **[Pathogenic]**	*DNAH5* GRCh38 5:13753289GA > G, NM_001369.3:10815del, p.D3605fs1 **[Pathogenic]**	Confirmed	Normal situs, no NRDS, persistent wet cough since birth, lower lobectomy age 10y, bronchiectasis, rhinitis since birth, no ear issues	30	Static	Static	ODA	DNAH5 absent
B	*DNAI1*	*DNAI1* GrCh38 9:34514436G > A, NM_012144.4:c.1612G > A, p.A538T **[Likely Pathogenic]**	Homozygous	Confirmed	*SI*, NRDS, Rhinitis, bronchiectasis, glue ear	2	Mostly static	Not done	ODA	Not done
C	*RPGR*	*RPGR* GRCh38 X:38317333 T > C NM_000328.3:c.602A > G, p.H201R **[VUS]**	Hemizygous	Possible	Normal situs, NRDS, persistent wet since early infancy, persistent rhinitis since early, glue ear. NO EYE SYMPTOMS	18	mixed length shorter and longer than usual, dyskinetic throughout	Cilia short, dyskinetic throughout	Normal	RPGR absent
D	*CFAP53*	*CFAP53 GRCh38* 18:50262051G > A, NM_145020.5:c.238C > T, p.R80X (no parental info)	*CFAP53* GRCh38 18:50242969C > A, NM_145020.5:c.1144G > T, p.E382X (no parental info)	Candidate	*SI*, no NRDS, recurrent chest infections started in adulthood, no ear issues, daily rhinitis	171	normal 2014			
E	*CEP164*	*CEP164* GRCh38 11:117411859C > T, NM_014956.5:c.4228C > T, p.Q1410X. Paternal	*CEP164* GRCh38 11:117387204C > T, NM_014956.5:c.1726C > T, p.R576X. Maternal	Candidate	Accompanied by father, BMI 28 103 kg, PICADAR 4; no NRDS but did have pnumonia as an infant; lifelong cough bronchiectasis, rhinitis	164	Reduced amplitude; some cilia long;		Within normal limits	

ACMG variant classification for each variant is given in bold

The table provides case ID, gene and variant details (with GRCh38 genomic coordinates) and classification (following ACMG guidelines) and final diagnosis. Clinical information provided includes clinical history, nasal nitric oxide levels (nNO (nL/min)), high-speed video (HSV) microscopy analysis from fresh nasal brushings and afterculture of these cells at air–liquid interface (ALI) transmission electron microscopy (TEM) and immunofluorescence (IF) microscopy findings. SI = *Situs inversus*, NRDS = neonatal respiratory distress syndrome, ODA = outer dynein arm defect

The *DNAI1* patient had a typical clinical history (*situs inversus*, neonatal respiratory distress, daily wet cough, perennial rhinitis, bronchiectasis and glue ear). This patient was referred for PCD diagnostics, which was typical for a person with *DNAH1* disease (low nNO, static cilia on HSVM and TEM showed outer dynein arm defects). Similarly the *DNAH5* patient had a classical clinical history and diagnostic findings (low nNO, static cilia before and after culture at air–liquid interface (ALI), TEM showed outer dynein arm defects and IF showed absence of DNAH5 from the cilium).

Whilst review of the *RPGR* patient’s clinical history was typical for PCD, an ophthalmologist confirmed the patient’s description of no eye disease, despite the association of *RPGR* variants with X-linked retinitis pigmentosa. This patient subsequently underwent PCD diagnostics, which showed a low nNO, abnormal HSVM before and after culture at air–liquid interface (ALI), dyskinetic throughout. TEM showed normal cilia ultrastructure, but IF imaging showed absence of RPGR from the cilium (Fig. 6). The patient was male and the variant was a hemizygous RPGR missense p.(His201Arg), absent from gnomAD, predicted by Polyphen to be probably damaging (score 0.996) and by SIFT to be deleterious (score 1).

These cases demonstrate how even in large bronchiectasis clinics, cases of PCD can be missed, and in these cases WGS can be invaluable for genetic diagnosis. The *RPGR* case further confirms the contribution of *RPGR* variants in PCD and provides some evidence that the *RPGR* PanelApp rating should be reviewed as it has been down-graded in the current PCD panel.

In the remaining unsolved non-CF bronchiectasis cases we did not find any untiered SNVs or SVs in PCD genes so we applied a functional candidate gene approach by using an updated version of the Syscilia Gold Standard list of known cilia genes [23] and CiliaCarta list of candidate cilia genes [24]. This identified biallelic variants in two cilia or centrosomal genes which have been linked to other OMIM diseases but not PCD (Table 4, Fig. 5).

One individual had biallelic *CFAP53* stop gain variants which segregated within the family. *CFAP53* variants have been associated with visceral heterotaxy type 6 in patients [37, 38] but the gene has not been previously described in PCD. Whilst this protein is present along the axoneme of cilia on human nasal respiratory epithelial cells, motility defects have not been described in the respiratory cilia in patients with *CFAP53* mutations [37]. However, recent work in mice has shown that tracheal and ependymal cilia show an altered beat pattern in *Cfap53* mutant mice [39]. In these mice the 9 + 0 nodal cilia (required for left–right patterning) are completely immotile, but the 9 + 2 motile cilia have subtle defects in the beat pattern. The respiratory phenotype of these mice was not described. Clinical review of our patient showed *situs inversus*, no NRDS, recurrent chest infections which started in adulthood, no ear issues, daily rhinitis, normal nNO and cilia which appeared normal on a historical HSVM of nasal epithelium (2014). We aim to repeat HSVM on a primary nasal epithelium brushing at the earliest opportunity. We suggest that biallelic mutations in *CFAP53* cause congenital *situs* defects, but may also be responsible for subtle respiratory cilia defects manifesting as adult-onset respiratory problems, although this will require further investigation since the lung disease may have a different aetiology. *CFAP53* has not currently been considered in the PCD panel in PanelApp but several other CFAP genes are rated amber in the PCD panel.

The other individual had compound heterozygous stop gain variants in *CEP164.* nNO was in the normal range, but HSVM analysis showed reduced amplitude of ciliary beating and some long cilia, with 19% of cilia showing microtubular disorganisation, mostly rearrangements. *CEP164* is not recognised as a PCD gene, but has been reported in a case of dual Bardet-Biedl syndrome (BBS) and PCD presentation [40] and our patient had some features of BBS. We are investigating this further as a research case.

## Discussion

Our data show that WGS testing in a clinically well-characterised PCD cohort has a high pick-up rate (16/21 patients received confirmed (n = 9)/probable (n = 4)/possible (n = 3) diagnosis) although 12/16 of these could have been picked up by current gene panel testing (Genomic Medicine Service PCD panel (R189), 46 green genes). Most cases were recruited and sequenced as trios (proband and parents), which was generally not required, as a diagnosis could have been achieved by singleton analysis with targeted sequencing of parents to determine phase in a small subset of cases. This suggests that WGS was useful but not cost-effective in 13/16 cases. WGS was chosen over WES in the 100,000 Genomes Project in an attempt to identify which conditions and cases most benefit from WGS over WES in clinical diagnostics, and to provide a large dataset of WGS data for future research. Our study suggests that in the case of PCD, WGS has little advantage over gene panel testing, except in unresolved cases where gene panel testing has not provided genetic diagnoses. In our opinion, gene panel testing is a more sensible first line of testing, followed by WES in unresolved cases. The impact of the 100,000 Genomes Project for PCD testing is that it confirms the efficiency and cost effectiveness of gene panel testing. WGS will unlikely become a frontline diagnostic test for PCD, even in the most well-resourced healthcare systems, but remains an extremely valuable research tool.

In 4/16 diagnoses WGS provided results which would have been missed by gene panel testing, through detection of SVs *in trans* with pathogenic/likely pathogenic coding SNVs. In some cases these coding SNVs were detected by prior gene panel testing but SV/intronic variants were not detected. 3 of these 4 diagnoses in the PCD cohort were obtained through research analysis of unsolved patients involving extraction of heterozygous SNVs followed by analysis of intronic variants and SVs. The current Genomics England Ltd clinical analysis pipeline does not tier deep intronic SNVs, nor SVs < 10 kb, and is not well optimised to detect cases of SNVs and SVs *in trans*. Heterozygous SNVs are not tiered in recessive disease, and SVs < 10 kb are not tiered by Genomics England Ltd yet 3 of the 4 diagnostic SVs that we found were < 10 kb. The amount of variant data generated by WGS requires filtering, but this needs to be done very carefully to avoid losing relevant variants. Our findings suggest that heterozygous SNVs should be tiered to maximise diagnostic return in clinically well-defined recessive diseases with established genotype/phenotype correlations such as PCD, and SVs should be tiered alongside SNVs in the Genomics England Ltd clinical analysis pipeline.

The identification of a heterozygous *CCDC114* splice donor site variant alongside a novel deep intronic VUS highlights that we are likely missing diagnoses by continuing to focus on coding regions in our variant analyses despite sequencing the whole genome. In order to more meaningfully study non-coding regions and synonymous changes we need better tools to predict the effect of such changes on splicing, and allow effective filtering of these. Unless intronic sequences can be screened efficiently and accurately, then WGS may have little advantage over WES (beyond permitting more accurate SV analysis). SpliceAI is a tool with higher sensitivity (0.8987) and specificity (0.9162) for predicting the effect of genetic variants on splicing than any previous tool, but still has false positive and false negative rates of around 10% [41]. We need to integrate RNA analyses into diagnostics to effectively pick up pathogenic splice variants, but this is difficult in a disease such as PCD in which the affected genes are only expressed in a few tissues of the body, which can’t always be easily sampled.

Two of the patients who received a ‘confirmed diagnosis’ of PCD based on SNVs in known PCD genes had previously received ‘probable diagnosis’ on the basis of the same variants found in prior gene panel testing. This reclassification of probable to confirmed diagnosis could have been achieved independently with re-review of existing data and targeted gene sequencing in parents. In cases where two variants in a known disease gene have been identified but one or both are VUS re-review of panel/WES data is more useful than further sequencing by WGS, especially if the VUS is in combination with a pathogenic/likely pathogenic variant and phenotype data support the gene of interest. It is clear that re-review of prior testing results is useful to improve diagnoses, and this process would greatly benefit from a motile cilia disease-specific public resource for depositing clinical variant data from PCD patients, similar to CFTR2 (https://cftr2.org/), a public repository for reporting CFTR variants found in CF patients. The BEATPCD consortium is currently working on such a resource, called ‘CilioVar’ (https://beat-pcd.squarespace.com/working-groups-summary). As a frontline test, WGS provides much greater scope for generating new diagnoses than WES or gene panel testing, as iterative re-analysis would include newly identified disease genes. However, WGS reanalysis brings resource (cost, person hours) implications for storing and analysing large volumes of data. Furthermore, the efficient use of resources also needs to be considered to balance analytical work with diagnostic yield. Although the standardised Genomics England Ltd WGS data analysis pipeline has made significant steps towards streamlining and automating the process of prioritisation of variants likely to be clinically significant, the requirement to carry out expert clinical variant classification to confirm diagnoses remains the rate-limiting step for the implementation of large-scale WGS. Unfortunately, this was poorly resourced in the 100,000 Genomes Project, leading to delays in return of results. It is clear from the 100,000 Genomes Project that adequate funding of data analysis and interpretation is a critical and indispensable component for delivering a WGS-based diagnostic service.

One of the major advantages of WGS over gene panel testing is that it provides significant opportunities for research and novel disease gene discovery. In our study WGS was especially useful in PCD variant negative cases allowing potentially pathogenic variants in candidate genes to be identified in four cases, although these remain to be functionally investigated and proven to be pathogenic. However, for conditions with defined phenotypes and high pickup rates like PCD, fixed panels still have diagnostic utility, so that the more expensive WGS can be reserved for more heterogeneous conditions such as intellectual disability (> 1000 known genes) where gene agnostic approaches can be used.

Finally, WGS was useful in helping to confirm that 2/21 patients in the PCD cohort had no genetic evidence to support a diagnosis of PCD. Negative genetic findings prompted these 2 patients to be re-reviewed clinically, suggesting that they are actually unlikely to have PCD. This highlights how difficult PCD can be to diagnose, even in specialist PCD diagnostic centres. The *DRC1* case we present demonstrates the important role that genetic testing plays as an additional diagnostic test in cases of PCD where patients do not have a strong history (for example, low PICADAR score [34] and normal nNO). The *DCR1* patient was aged 34 years at the time of referral for diagnostic testing, had a PICADAR score of 4/14 which is below the level recommended for referral for diagnostic testing and, unlike many with *DRC1* mutations, had normal nitric oxide levels. They therefore risked not proceeding to further diagnostic testing and even following referral, PCD can erroneously be discounted if subtle defects in ciliary beat pattern and TEM are not detected. Cilia structure may appear grossly normal under TEM [13], but further inspection often shows central apparatus abnormalities or microtubule disorganization in a proportion of cilia [17]. The diagnosis in this individual demonstrates the value and importance of careful high-speed video imaging analysis and TEM analysis in suspected cases of PCD, and how genetic testing can help to confirm this analysis. Although this genetic diagnosis could have been identified through gene panel testing and WGS was useful but not essential, we wished to highlight this difficult diagnostic case, and how a diagnosis was obtained.

Alongside 16 genetic diagnoses in the PCD cohort, and identification of candidate genes in 2 additional patients in the PCD cohort, we also obtained genetic diagnosis of PCD in 3 patients in the non-CF bronchiectasis cohort and identified candidate motile ciliopathy genes in 2 additional patients in this cohort. In contrast to the PCD cohort, the non-CF bronchiectasis cohort comprised adult patients, generally recruited as singletons. Whilst this would not have posed an issue in the PCD cohort, in non-CF bronchiectasis, which is a genetically heterogenous group of disorders, with different patterns of inheritance and some likely non-genetic causes, the difficulty in segregating variants without family member’s sequence, and lack of clarity of the pattern of inheritance made analysis more difficult. It would be advantageous to recruit these patients for genetic testing with family members, where possible. These findings show that even in large adult bronchiectasis clinics, the diagnosis of PCD may be overlooked (entered in as bronchiectasis rather than PCD). Therefore, there are likely to be many patients with PCD in adult pulmonology clinics- if clinicians do not revisit the early life history, they may miss patients with a history suggesting a genetic cause. The identification of candidate gene variants in 2 patients in the non-CF bronchiectasis cohort suggests that novel disease genes may be discovered by investigating cases with atypical PCD and that the phenotypic spectrum of PCD may be broader than previously described.

Finally, we present a case of PCD without ophthalmological involvement, associated with a hemizygous variant in *RPGR* case to provide further evidence that this gene can be a cause of PCD. To be rated green on PanelApp pathogenic variants need to be identified in ≥ 3 independent families, so identification and publication of additional novel cases in rare/amber genes is important.

## Conclusion

PCD is a difficult condition to diagnose as there is no “gold standard” diagnostic tool. Genetic testing is useful to confirm a diagnosis of PCD, especially in cases which do not have a typical history, such as in the *DRC1* case we present.

PCD diagnoses continue to be missed even in large adult bronchiectasis centres. WGS in adults with non-CF bronchiectasis is useful for detecting missed diagnoses of PCD in this group of patients, and trio analysis is useful in this cohort.

WGS is a useful diagnostic test in a PCD cohort but is only an effective use of resources where patients have received no findings or have only had heterozygous variants found from prior gene panel testing. A significant proportion of diagnoses were missed through the automated Genomics England Ltd tiering pipeline, and were only picked up with additional labour-intensive manual analysis of patient’s genome data.

Proband-only gene panel/WGS testing followed by targeted testing of parents where required is sufficient for diagnosis of PCD in most cases, and is more cost-effective.

Heterozygous SNVs and SVs from WGS should not be filtered out of analysis in clinically well-defined recessive diseases with established genotype/phenotype correlations such as PCD.

WGS is a powerful tool for gene discovery. We identify 2 novel candidate PCD genes in our PCD cohort and another 2 in our non-CF bronchiectasis cohort.

The advantages of WGS over gene panel testing will be more apparent when we have better tools for intronic variant filtering and automated systems for clinical variant classification and periodic re-review of data.

## Supplementary Information

**Additional file 1. Table 1**: Genes on 19 and 29 gene next generation sequencing panel used for PCD genetic testing in patients in the Wessex PCD cohort prior to WGS through the 100,000 Genomes Project

**Additional file 2.** Additional information on 100,000 Genomes Project recruitment, sequencing, sequence analysis and variant filtering and tiering performed by Genomics England Ltd.

## Data Availability

The data that support the findings of this study are available from Genomics England Ltd but restrictions apply to the availability of these data, which were used under license for the current study, and so are not publicly available. For further details contact gecip-help@genomicsengland.co.uk. For any other queries contact g.wheway@soton.ac.uk or jlucas1@soton.ac.uk.

## Abbreviations

ACMG: American College of Medical Genetics
ALI: Air–liquid interface
AMP: Association for Molecular Pathology
CF: Cystic fibrosis
GMC: Genome Medicine Centre
HSVM: High speed video microscopy
IGV: Integrative Genomics Viewer
MTD: Microtubular disorganisation
N-DRC: Nexin-dynein regulatory complex
nNO: Nasal nitric oxide
NRDS: Neonatal respiratory distress syndrome
PCD: Primary ciliary dyskinesia
SNV: Small nucleotide variant
SV: Structural variant
TEM: Transmission electron microscopy
VUS: Variant of uncertain clinical significance
WES: Whole exome sequencing
WGS: Whole genome sequencing
