# Supplementary material for: Whole genome sequencing in the diagnosis of primary ciliary dyskinesia
Source: BMC Med Genomics. 2021 Sep 23;14:234. doi: 10.1186/s12920-021-01084-w (PMC8461892; doi:10.1186/s12920-021-01084-w)
Supplement: Supplementary file 1 — Additional file 1. Table 1: Genes on 19 and 29 gene next generation sequencing panel used for PCD genetic testing in patients in the Wessex PCD cohort prior to WGS through the 100,000 Genomes Project [file 12920_2021_1084_MOESM1_ESM.docx]

| **19 gene panel** | **29 gene panel** |
| --- | --- |
| *CCDC103* | *ARMC4* |
| *CCDC39* | *C21orf59* |
| *CCDC40* | *CCDC103* |
| *DNAAF1* | *CCDC114* |
| *DNAAF2* | *CCDC151* |
| *DNAAF3* | *CCDC39* |
| *DNAAF5* | *CCDC40* |
| *DNAH11* | *CCDC65* |
| *DNAH5* | *CCNO* |
| *DNAI1* | *DNAAF1* |
| *DNAI2* | *DNAAF2* |
| *DNAL1* | *DNAAF3* |
| *DYX1C1 (DNAAF4)* | *DNAAF5* |
| *HYDIN* | *DNAH11* |
| *LRRC6* | *DNAH5* |
| *RPGR* | *DNAI1* |
| *RSPH4A* | *DNAI2* |
| *RSPH9* | *DNAL1* |
| *NME8* (Red on PanelApp) | *DRC1* |
|  | *DYX1C1 (DNAAF4)* |
|  | *HYDIN* |
|  | *LRRC6* |
|  | *RPGR* |
|  | *RSPH1* |
|  | *RSPH3* |
|  | *RSPH4A* |
|  | *RSPH9* |
|  | *SPAG1* |
|  | *ZMYND10* |

**Supplementary Table 1.** 19 and 29 gene PCD panel used in prior standard of care testing
