# Supplementary material for: Whole genome sequencing in the diagnosis of primary ciliary dyskinesia
Source: BMC Med Genomics. 2021 Sep 23;14:234. doi: 10.1186/s12920-021-01084-w (PMC8461892; doi:10.1186/s12920-021-01084-w)
Supplement: Supplementary file 2 — Additional file 2. Additional information on 100,000 Genomes Project recruitment, sequencing, sequence analysis and variant filtering and tiering performed by Genomics England Ltd. [file 12920_2021_1084_MOESM2_ESM.docx]

**Supplementary Information**

Recruitment to the 100,000 Genomes Project was initially restricted to patients who had undergone prior genetic diagnostic testing and not received a genetic diagnosis, although later in the project this was relaxed to include patients who had not undergone previous genetic testing. The hybrid research and diagnostic project involved recruitment and consenting of patients, collection of blood (plus tumour tissue in the case of cancer patients), and recording of Human Phenotype Ontology (HPO) terms describing the patient’s phenotype. DNA was extracted and stored in a central bioresource, PCR-free libraries prepared and sequenced using Illumina short-read technology, sequence data aligned initially to human Genome Build 37, and more recently to Build 38, followed by variant calling and annotation all carried out by Illumina. Genomics England Ltd (GEL) performed variant filtering through a clinical analysis pipeline, to identify potentially diagnostic small nucleotide variants, which were “tiered” according to likelihood of being the pathogenic cause of disease if consistent with the observed pattern of inheritance (1).

Whilst the whole genome was sequenced in each individual, only selected regions of the genome were analysed in the GEL clinical analysis pipeline. Virtual Gene Panels were created using an application called PanelApp, a crowdsourcing tool in which clinicians, clinical scientists and academic researchers contributed to the rating of genes in terms of their diagnostic value (2). Primary ciliary disorders (**Table 1**) and non-CF bronchiectasis (**Table 2**) are distinct gene panels. Genes were assigned colours based on a traffic-light system; green representing confidently diagnostic genes, amber representing genes where there is reasonable evidence of pathogenicity associated with a particular clinical condition but insufficient to be robustly diagnostic, and red representing genes where there is limited evidence of pathogenicity associated with a particular clinical condition, insufficient to be robustly diagnostic. Based on HPO terms assigned to a patient by the recruiting clinician, virtual panels were assigned to the analysis pathway by automated Panel Assigner. While families were recruited under specific clinical indications, such as PCD or non-CF bronchiectasis the panel(s) applied were determined by the HPO terms recorded at recruitment. Consequently, the primary ciliary disorders panel (**Table 1**) was applied to some but not all non-CF-bronchiectasis cases. Conversely, the primary ciliary disorders panel was also applied to many cases with other clinical indications.

The GEL clinical analysis pipeline then involved filtering variants through a custom pipeline and prioritising them for clinical assessment using a “tiering” system (**Figure 1**). In brief, single nucleotide variants (SNVs) or small insertions or deletions (indels) were filtered to exclude common variants and only retain variants which are rare in the population for analysis. Synonymous variants were excluded, variants which did not follow the relevant mode of inheritance of the genes in the selected PanelApp gene panel were excluded (ie monoallelic changes in recessively inherited disease genes were excluded) and, where multiple family members were recruited any variant which didn’t segregate appropriately within the family was excluded. Variants were then assigned Tier 1 if they were protein truncating (stop gain, frameshift, splice donor or splice acceptor) variants or any *de novo* variant in a green gene on a PanelApp gene panel assigned to the participant, Tier 2 if they were protein altering variants (missense or splice region) in a green gene on a PanelApp gene panel assigned to the participant, and tier 3 for any variant in any other gene. In practical terms for PCD, the HPO terms entered resulted in the PCD gene panel being applied for every participant and rare biallelic SNVs and indel variants in known PCD genes were tiered as tier 1 or 2, and variants in any other gene were tiered as tier 3. In the case of autosomal recessive PCD, monoallelic variants were not tiered. This presents potential for missed diagnosis in cases of compound heterozygous SNV and SV variants in known disease genes.

In the clinical GEL analysis, SV analysis of sequence data, based on counting sequence reads, was implemented in February 2019, so that SV analysis was applied to only a third of all Wessex families recruited. SVs <10kb were excluded by the analysis filters. If a SV involved a green gene in one of the panels applied it was included in Tier A; all other SVs >10 kb were in Tier Null. Different to the tiering of SNVs, tiering of SVs did not take into account the inheritance pattern of the gene, therefore single SVs in recessive genes were tiered and the corresponding sequence data could be manually interrogated for the presence of a sequence variant.

Recruitment was coordinated by 13 Genome Medicine Centres (GMCs) across England, with tiered variant data being reported back to GMCs for review. GMCs were expected to review all Tier 1 and Tier 2 sequence variants and Tier A SVs, in line with ACMG guidelines (3) and subsequently report back to recruiting clinicians and patients, often over a timeline longer than most patients and clinicians anticipated at the start of the project, due to lack of resources provided to GMCs for variant review and reporting (4).

In parallel, all anonymised sequence data, variant call format (VCF) files, structural variant files, HPO data and tiering data was made available for interrogation by members of Genomics England Clinical Interpretation Partnerships (GeCIPs) who access anonymised data through the secure online Research Environment within the Genomics England Data Embassy. PCD and non-CF bronchiectasis research analysis has been conducted as part of the Paediatric and Respiratory GeCIPs.

1. Turnbull C, Scott RH, Thomas E, Jones L, Murugaesu N, Pretty FB, et al. The 100 000 Genomes Project: bringing whole genome sequencing to the NHS. BMJ. 2018;361.

2. Martin AR, Williams E, Foulger RE, Leigh S, Daugherty LC, Niblock O, et al. PanelApp crowdsources expert knowledge to establish consensus diagnostic gene panels. Nat Genet. 2019;51(11):1560-5.

3. Richards S, Aziz N, Bale S, Bick D, Das S, Gastier-Foster J, et al. Standards and guidelines for the interpretation of sequence variants: a joint consensus recommendation of the American College of Medical Genetics and Genomics and the Association for Molecular Pathology. Genet Med. 2015;17(5):405-24.

4. Moss C, Wernham A. The 100 000 Genomes Project: feeding back to patients. BMJ. 2018;361.
